# Global myocardial work parameters measured by the index beat method are comparable to the average of 10 beats in patients during atrial fibrillation

**DOI:** 10.3389/fcvm.2025.1612962

**Published:** 2025-08-26

**Authors:** Ling-Yun Kong, Xiu-Juan Wang, Ling-Ling Chen, Wei Xiang, Fang Liu

**Affiliations:** Department of Cardiovascular Disease, Beijing Tsinghua Changgung Hospital, School of Clinical Medicine, Tsinghua Medicine, Tsinghua University, Beijing, China

**Keywords:** atrial fibrillation, left ventricular systolic function, index beat, single beat, myocardial work

## Abstract

**Introduction:**

Evaluation of left ventricular (LV) global systolic function is clinically important for patients with atrial fibrillation (AF); however, the rhythm irregularity inherent to AF poses challenges for assessing novel LV systolic function parameters, such as global myocardial work (MW). This study aimed to validate the feasibility of using the single index beat method to quantify LV MW during echocardiography in patients with AF, compared with the traditional 10-beat average method.

**Methods:**

A prospective study was performed in 120 patients with AF at the time of the index echocardiography. Global longitudinal strain was assessed using speckle tracking echocardiography from a triplane dataset, followed by MW analysis to calculate global myocardial work index (GWI), global constructive work (GCW), global wasted work (GWW), and global work efficiency (GWE). A total of 10 consecutive beats were evaluated, with both the average value and the maximal difference among the 10 beats recorded. The index beat was defined as on in which the ratio of the preceding to the pre-preceding R-R interval was approximately 1 (0.96–1.04). MW parameters from the index beat were extracted for analysis. Inter-method consistency was assessed using the intra-class correlation coefficient (ICC) with a single-rater, absolute agreement, two-way random effects model. Inter- and intra-observer reproducibility was also assessed.

**Results:**

Global MW derived from the index beat was comparable with the average of 10 beats: GWI, 1,157.19 ± 416.83 vs. 1,188.98 ± 452.96 mmHg% (*p* < 0.05); GCW, 1,721.46 ± 524.69 vs. 1,732.46 ± 524.24 mmHg% (*p* > 0.05); GWW, 237.95 (183.60) vs. 207.50 (207.25) mmHg% (*p* < 0.001); and GWE, 85.80% (11.05) vs. 86.50% (12.75) (*p* < 0.001). Consistency analysis showed that ICCs for all assessed MW parameters were >0.87. Satisfactory inter- and intra-observer reproducibility of the measurements by the index beat method was also found.

**Conclusions:**

Global MW measured using the index beat method demonstrated good agreement with the average over 10 beats in patients with AF, supporting its reliability as a surrogate for the traditional method in clinical practice.

## Introduction

Atrial fibrillation (AF) is currently the most common sustained arrhythmia (1). It is associated with increased cardiovascular mortality and morbidity (2), and accurate evaluation of left ventricular (LV) global systolic function using echocardiography is clinically significant (3). However, it is also challenging due to the constantly changing LV contractile ability with varying cardiac cycle length that is characteristic of AF (4). Previously, LV global longitudinal strain (GLS) measured across 17 segments using two-dimensional (2D) speckle tracking echocardiography (STE) has been validated as a more reliable and sensitive parameter of LV systolic dysfunction than single-sectional GLS or traditional LV ejection fraction (EF) (5–7). More recently, non-invasive global myocardial work (MW) parameters derived from overall GLS have been proposed to overcome the limitation of strain's dependence on afterload (8), and have demonstrated better diagnostic and prognostic ability compared with GLS (9, 10). AF has been associated with subclinical myocardial dysfunction, as demonstrated by myocardial work parameters in a population-based cohort of AF patients compared with healthy individuals (11, 12). However, existing literature on the use of MW in AF remains limited, primarily due to the beat-by-beat variability of LV systolic function. Nowadays, triplane echocardiography allows real-time demonstration of three apical views simultaneously, making it possible to obtain a reliable overall GLS for each heartbeat in AF. Given that MW is a derivative of GLS, it is reasonable to expect its feasibility in this setting. Current guidelines acknowledge the inter-beat variability in LV systolic performance during AF and recommend averaging at least five beats for quantification as the standard method (13), with the use of a “‘representative beat”‘ considered only “‘acceptable.”‘ However, growing evidence supports the reliability of the index beat method for evaluating LV systolic function in AF (14–17). Therefore, the present study aimed to validate the reliability of the index beat method compared with the average-beat method for assessing LV MW in patients with AF using triplane echocardiography.

## Methods

### Study population

**Figure 1:**
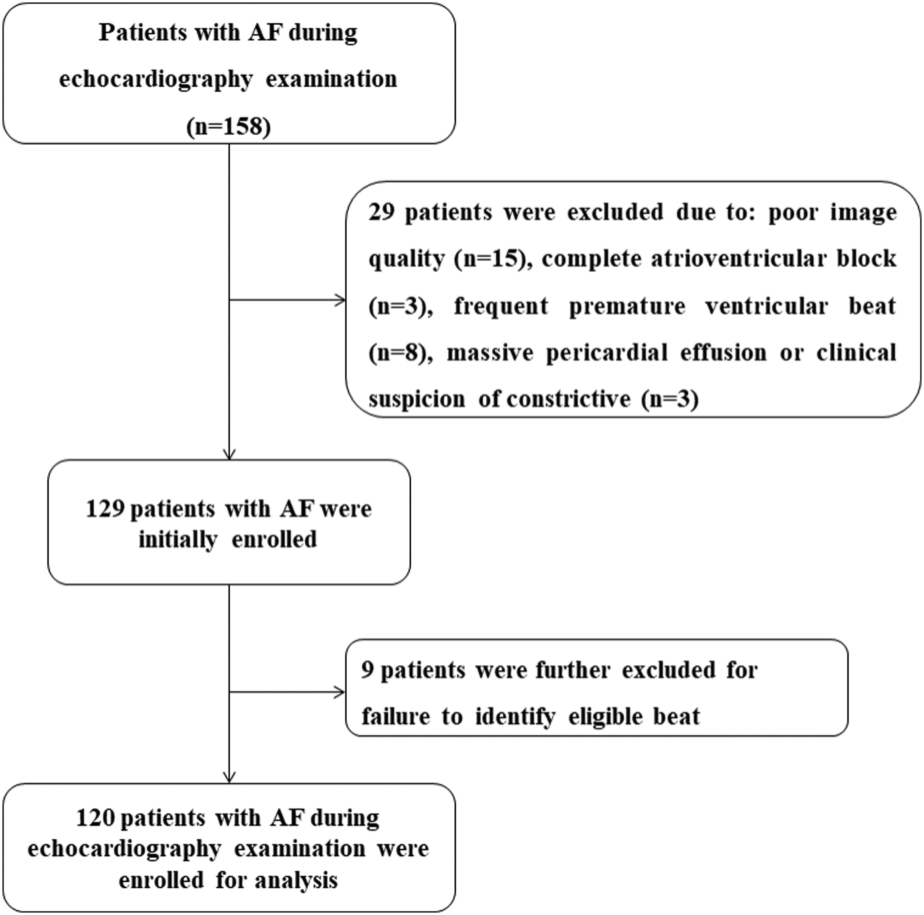
Flowchart of patient enrollment.

A prospective observational study was conducted between June 2021 and June 2024 (ChiCTR2100050725). A total of 158 consecutive patients in AF during echocardiographic examination were initially enrolled according to the pre-specified protocol, regardless of AF type or duration. Rhythm was confirmed by a simultaneously recorded surface electrocardiogram and supported by a single positive peak of the diastolic mitral flow spectrum with variable duration, velocity range, and intervals. Exclusion criteria were as follows: more than two non-visualized segments (*n* = 15), complete atrioventricular block (*n* = 3), frequent premature ventricular beats (*n* = 8), and massive pericardial effusion or clinical suspicion of constrictive pericarditis (*n* = 3). Upon analysis, patients were also excluded if no beat met the definition of the index beat (*n* = 9). Figure 1 shows the patient enrollment flowchart. To enhance real-world applicability, the exclusion criteria were kept narrow; thus, patients with structural heart disease, including severe valvar disease, congenital heart disease, or reduced EF, were included. In total, 120 AF patients were finally enrolled. The study was approved by our institutional review board (reference no. 21281-0-02) and written informed consent was obtained from all participants.

### Clinical data

Patient demographics and clinical characteristics were recorded. All medications remained unchanged during the study.

### Echocardiography

**Figure 2:**
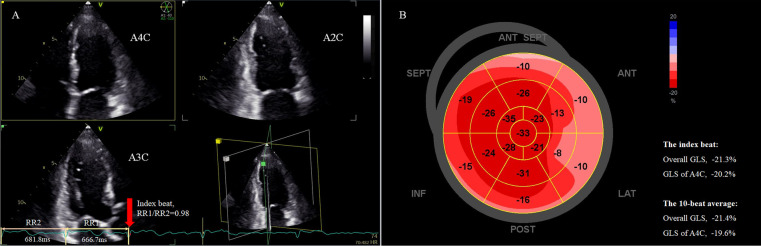
Assessment of LV overall GLS in a patient with persistent AF using the index beat. **(A)** Strain analysis was performed based on triplane echocardiography showing simultaneous apical two-, three-, and four-chamber views. The index beat (red arrow) was defined as the beat with an almost equal preceding (RR1) and pre-preceding (RR2) R-R intervals. **(B)** The bull's-eye plot of regional GLS. The overall GLS value and GLS of the atrial four-chamber view of the index beat closely matched the 10-beat average value. AF, atrial fibrillation; GLS, global longitudinal strain; LV, left ventricle.

Patients were scanned in the left supine position with a commercially available ultrasound system (Vivid E9; GE Vingmed Ultrasound AS, Horten, Norway). All patients underwent comprehensive 2D echocardiography (2DE) with a M5S transducer (1–5 MHz), followed by triplane echocardiography with a 3 V transducer (1.7–3.3 MHz). Brachial blood pressure was measured after examination (averaged over three measurements). With the M5S transducer, standard 2D, color, pulsed, and continuous wave Doppler images were acquired according to the guidelines (18). LV apical two-, three-, and four-chamber views were sequentially and respectively acquired. With 3 V probe and the triplane echocardiography mode, apical two-, three-, and four-chamber views focusing on LV was demonstrated simultaneously in one ultrasonic view (Figure 2A). Care was taken to ensure that all 17 LV segments were visualized without apical foreshortening, and the image frame rates were maintained in the range of 40–80 Hz. At least 10 cycles were stored for analysis, consistent with previously described methodology (17).

Conventional 2DE measurements were performed by a single expert echocardiographer (L-YK) following the guidelines (18). LV volume, EF, and left atrial volume were measured using the uniplane Simpson's method from the apical four-chamber view only, as myocardial contractility varied beat-by-beat and biplane summation could not accurately represent the true systolic status of any beat (4, 19).

STE analysis was performed using a commercially available software (EchoPAC V204, GE). Strain was calculated as the percentage change in end-systolic length/initial length. GLS was measured from standard apical two-, three-, and four-chamber views of the triplane dataset (Figure 2). The bull’s eye of segmental peak longitudinal strain with sectional GLS, overall GLS of 17 segments, and heart rate for each beat could be obtained (Figure 2B). Measurements were performed beat-by-beat for 10 consecutive beats, and mean apical four-chamber GLS and overall GLS over these 10 beats were calculated for analysis, as these parameters are most frequently used in clinical practice. For each heartbeat, the R-R interval (ms) was calculated as 60,000 divided by the heart rate (bpm), the latter obtained automatically from the analysis software. For example, if the heart rate for a selected cycle was 60 bpm, the R-R interval would be 60,000/60 = 1,000 ms. The index beat was defined as the beat with nearly equal preceding (RR1) and pre-preceding (RR2) intervals (Figure 2A). Because a ratio of RR1/RR2 exactly equal 1.0 is difficult to obtain in AF, we accepted a range of 0.96–1.04 as eligible (17). The GLS values for both the apical four-chamber view and overall GLS from the index beat were extracted for analysis. The echocardiographer performing the initial measurements was blinded to both the average MW over 10 beats and the index beat for each patient. An index beat-derived overall GLS was considered representative if its absolute difference from the 10-beat average was within 5%.

**Figure 3:**
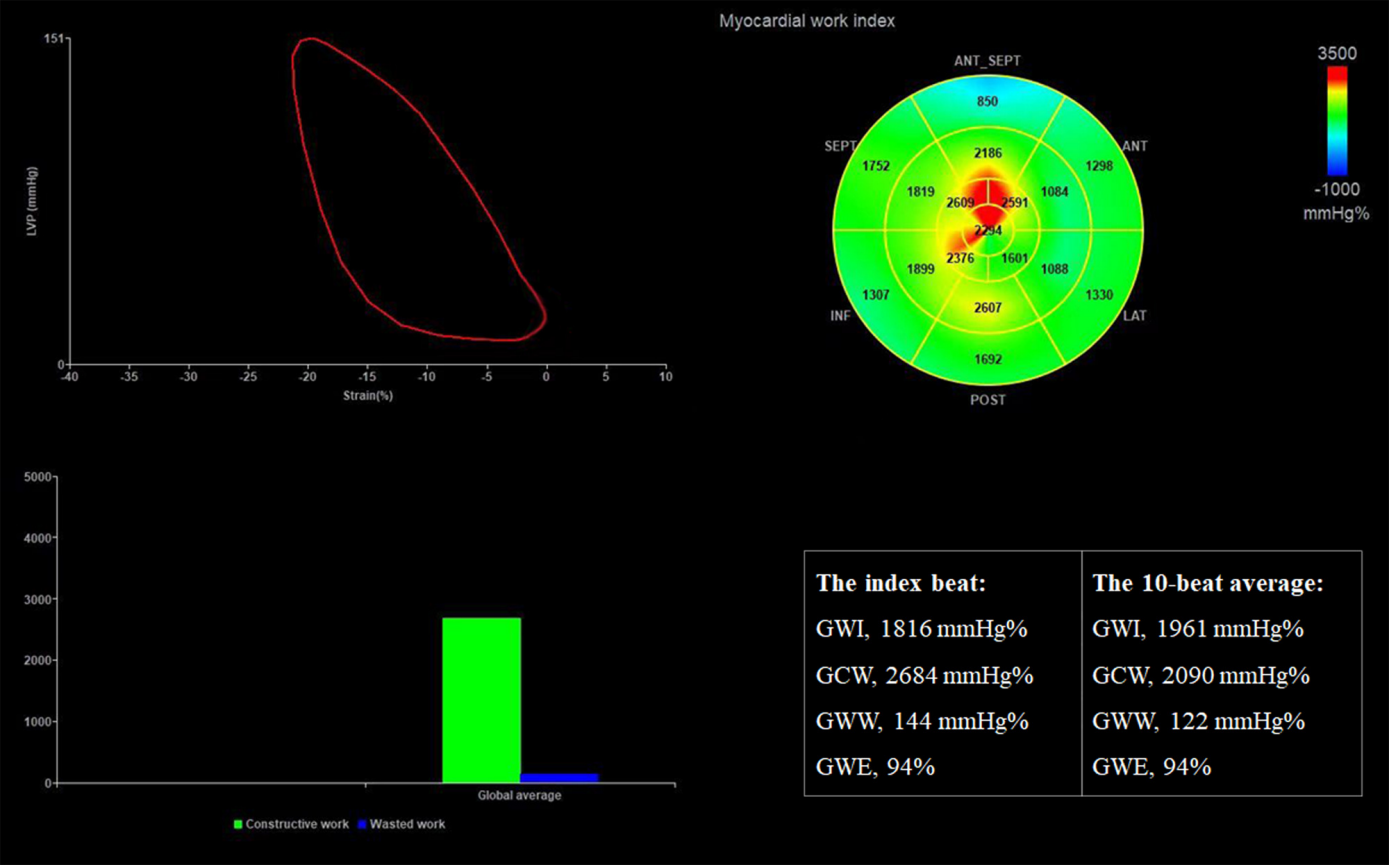
Assessment of LV MW parameters in the same patient. The results show that GWI, GCW, GWW, and GWE derived from the index beat are similar to or the same as the average value over 10 beats. GCW, global constructive work; GWE, global myocardial work efficiency; GWI, global myocardial work index; GWW, global wasted work.

Quantification of MW was performed using the same software package following each GLS. It was also evaluated beat-by-beat. Strain and pressure data were synchronized by aligning the valvular event times (20). The mitral and aortic valve opening and closure was determined from the parasternal long axis view. The result of MW analysis for each heartbeat was presented as a figure with four panels (Figure 3). A non-invasive pressure strain loop was then derived, with the right lower point indicating mitral closure when the strain value was 0. Its area was an index of global MW. Segmental LV MW values were displayed on a bull's eye plot. The global MW index (GWI) was calculated as the average of all 17 segments. Global constructive work (GCW) was defined as the sum of positive work generated by myocardial shortening during systole and negative work during isovolumic relaxation. Global wasted work (GWW) represented the energy loss from myocardial lengthening in systole and shortening during isovolumic relaxation. Global MW efficiency (GWE) was calculated as GCW divided by the sum of GCW and GWW (9).

### Reproducibility analysis

To assess the intra- and inter-observer reproducibility of the index beat method for assessing MW parameters, 24 individuals were randomly selected for re-analysis. Two independent observers (L-YK and X-JW) measured the same cine loops separately, and the same observer (L-YK) re-evaluated the images at least 4 weeks apart. The results were expressed as intra-class correlation coefficients (ICC) and 95% confidence intervals (CI). Excellent agreement was defined by an ICC > 0.75.

### Statistical analysis

Continuous variables are expressed as mean ± SD or median (inter-quartile range [IQR]), as appropriate. Categorical variables are presented as frequency (%). The Kolmogorov–Smirnov test was used to assess the normality of data distribution. Lin's Concordance Correlation Coefficient Power Analysis was used to estimate the sample needed. A minimum sample size of 101 subjects was estimated to achieve a power of 0.90, assuming a concordance correlation coefficient of 0.991. Differences between the values obtained by the index beat method and the average method were compared using paired Student's *t*-tests or Wilcoxon signed-rank tests, as appropriate. Agreement between the two methods was evaluated using the ICC (95% CI) based on a single-rater, two-way random effect model with absolute agreement. Bland–Altman analysis was not applied because the distribution of the differences between the two measurements was skewed. All clinical and echocardiographic data were analyzed using standard statistical software (SPSS 21.0 and MedCalc 15.0), and statistical significance was defined as *p* < 0.05.

## Results

### Patient characteristics and traditional echocardiographic parameters

The baseline demographic and echocardiographic characteristics of the patients are shown in Table 1. A total of 120 patients with AF during the initial echocardiographic examination were enrolled as the study population (63.33% men, mean age 71.30 ± 9.11 years). Of the patients, 102/120 (85.0%) had persistent AF. Most (69/120, 57.5%) patients fell in New York Heart Association functional class I. The median LVEF was 62.0% (14.0) (IQR 20%–76%). In total, 12 (10%) patients had LVEF ≤ 40%.

**Table 1:** Characteristics of the study population.

Variables	Participants (*N* = 120)
Demographics	
Age (years)	71.30 ± 9.11
Male	76 (63.33)
Clinical parameters	
BSA (m^2^)	1.77 ± 0.19
BMI (kg/m^2^)	24.98 ± 3.68
Pulse (bpm)	74.91 ± 14.24
Systolic blood pressure (mmHg)	126.72 ± 17.67
Diastolic blood pressure (mmHg)	79.38 ± 11.30
Type of AF, *N* (%)	
Paroxysmal AF	15 (12.50)
Persistent AF	102 (85.00)
Permanent AF	3 (2.50)
Status of cardiac function	
NYHA, *N* (%)	
Class I	69 (57.50)
Class II	31 (25.83)
Class III	16 (13.33)
Class IV	4 (3.33)
History of diseases, *N* (%)	
Hypertension	76 (63.33)
Diabetes	33 (27.50)
Heart failure	31 (25.83)
Mitral stenosis	16 (13.33)
Hypertrophic cardiomyopathy	4 (3.33)
Multiple myeloma	1 (0.83)
Coronary heart disease	23 (19.17)
Cancer	15 (12.50)
Hyperthyroidism	5 (4.17)

AF, atrial fibrillation; BSA, body surface area; BMI, body mass index; bpm, beats per minutes; NYHA, New York Heart Association.

### Myocardial strain and work

STE analysis was successfully performed in all patients. The median frame rate of traditional 2DE images and triplane echocardiography was 56.5 Hz (7.2 Hz) and 49.1 Hz (6.3 Hz), respectively. Baseline echocardiographic characteristics are listed in Table 2. It should be noted that among the 129 patients enrolled for STE analysis (Figure 1), as many as 43 (33.3%) patients did not present an index beat at the initial analysis. However, analysis of other cine loops from triplane datasets stored during the index examination helped find out 34 patients with at least one index beat, thus resulting in 9 (6.9%) patients excluded from further analysis. Among the 120 eligible patients, 46 (38.3%) had more than two index beats (35 patients had two, nine patients had three, and two patients had four index beats).

**Table 2:** Traditional echocardiogram parameters (*N* = 120).

Variables	Participants (*N* = 120)
Frame rate of 2DE	56.50 (7.20)
Frame rate of 3PE	49.10 (6.30)
Average index beat R-R (RR0) (ms)	731.71 (305.76)
Average pre-index beat R-R (RR1)	722.89 (195.28)
Index beat ratio of RR1/RR2	1.00 (0.04)
Left ventricle	
EDD (mm)	49.00 (8.50)
EDV (mL)	75.50 (34.50)
ESV (mL)	27.00 (22.00)
EF (%)	62.00 (14.00)
Left atrium	
Anteroposterior diameter (mm)	46.00 (10.00)
Axial diameter (mm)	65.50 (12.00)
Transverse diameter (mm)	53.00 (8.00)
LAVi-max (mL/m^2^)	93.50 (56.50)
LAVi-min (mL/m^2^)	74.00 (49.50)
Doppler echocardiography	
e'-Septal (cm/s)	8.00 (3.85)
e'-Lateral (cm/s)	12.00 (4.25)
E/e'	10.00 (5.80)
Moderate and above MR, *N* (%)	43 (35.83)
Moderate and above TR, *N* (%)	60 (50.00)

2DE, 2-dimensional echocardiography; 3PE, triplane echocardiography; EDD, end-diastolic dimension; EDV, end-diastolic volume; EF, ejection fraction; ESV, end-systolic volume; FAC, fraction area change; HR, heart rate; LAVi, left atrial volume indexed; MR, mitral regurgitation; TAPSE, tricuspid annular plane systolic excursion; TR, tricuspid regurgitation.

The median R-R interval of 10 beats was 753.28 ms (202.07 ms, range 485.9–1,268.5 ms) (Table 2), among whom 4 (3.3%) had a mean R-R interval <500 ms (range 485.9–496.5 ms), corresponding to a mean heart rate of 122–128 beats per min. The median R-R interval of index beat was 731.71 ms (305.76 ms, range 384.6–1,818.2 ms) (*P* = 0.87 compared with the average R-R of 10 beats). The median RR1/RR2 ratio of the index beat was 1.0 (IQR 0.4).

Table 3 shows the overall GLS and apical four-chamber GLS derived from the index beat closely approximated the corresponding 10-beat average values, with only small inter-method differences. These findings are consistent with those of a previous study on apical four-chamber GLS using the index beat method (15). The ICCs for GLS measurement by the two methods were also excellent (Table 4). Notably, only 1 (0.83%) of the 120 patients showed an index beat-derived GLS that differed from the average by more than 5% (index beat: −5.6% vs. mean: −10.79%). This patient had a wide GLS range, fluctuating from −19.8% to −4.0%, with R-R intervals varying from 377.36 to 1,000.00 ms. Among the 120 index beats analyzed, 7 (5.8%) had R-R intervals <500 ms (range 384.6–491.8 ms), of whom only 1 (14.3%) case had a non-representative GLS value.

**Table 3:** Results of and difference in the myocardial strain and work parameters between the two methods.

Variables	Average value	Index beat value	Inter-method difference	*P*-value	Maximal difference among the 10 beats
Myocardial strain					
GLS-A4C (%)	−14.17 ± 4.07	−14.40 ± 4.36	0.10 (1.70)	0.190	6.1 (3.1)
GLS-overall (%)	−13.93 ± 4.18	−14.14 ± 4.41	0.22 (1.13)	0.062	5.6 (2.8)
Myocardial work					
GWI (mmHg%)	1,157.19 ± 416.83	1,188.98 ± 452.96	−35.7 (173.53)	0.014*	575 (301.5)
GCW (mmHg%)	1,721.46 ± 524.69	1,732.46 ± 524.24	2.8 (134.03)	0.403	467 (207.75)
GWW (mmHg%)	237.95 (183.60)	207.50 (207.25)	21.95 (82.15)	0.001*	227 (217.25)
GWE (%)	85.80 (11.05)	86.50 (12.75)	−1.25 (3.95)	0.000*	11.5 (8)
HR (bpm)	83.10 (20.03)	82.00 (32.50)	0.85 (21.28)	0.349	46.5 (25.5)
R-R interval (ms)	753.28 (202.07)	731.71 (305.76)	4.87 (191.29)	0.869	434.81 (274.58)

A4C, apical four-chamber view; GCW, global constructive work; GLS, global longitudinal strain; GWE, global myocardial work efficiency; GWI, global myocardial work index; GWW, global wasted work; HR, heart rate.

* *P* < 0.05.

**Table 4:** Agreement analysis between the measurements by the two methods.

Variables	ICC (95% CI)	*P*-value
GLS-A4C	0.92 (0.89–0.95)	<0.001
GLS-overall	0.96 (0.94–0.97)	<0.001
GWI	0.93 (0.90–0.95)	<0.001
GCW	0.98 (0.96–0.98)	<0.001
GWW	0.87 (0.82–0.91)	<0.001
GWE	0.88 (0.82–0.92)	<0.001
HR	0.66 (0.55–0.75)	<0.001
R-R	0.60 (0.47–0.70)	<0.001

A4C, apical four-chamber view; CI, confidence interval; GCW, global constructive work; GLS, global longitudinal strain; GWE, global myocardial work efficiency; GWI, global myocardial work index; GWW, global wasted work; HR, heart rate; ICC, intra-class correlation coefficient.

For MW parameters, no differences were found in GCW measured using the two methods. The index beat method yielded slightly higher GWI and GWE, and slightly lower GWW, compared with the average method; however, these inter-method differences were small, especially when compared with the maximal differences observed among the 10 beats, which reflect random measurement variability (Table 3). Consistency analysis demonstrated excellent agreement between methods for MW parameters, with all ICC values at >0.75 (Table 4).

### Reproducibility analysis

For intra-observer repeat assessment of overall GLS, GWI, GCW, GWW, and GWE, ICC was 0.987 (95% CI, 0.971–0.994), 0.876 (95% CI, 0.720–0.945), 0.981 (95% CI, 0.957–0.992), 0.852 (95% CI, 0.660–0.936), and 0.904 (95% CI, 0.778–0.958), respectively (all *P* = 0.000). For inter-observer repeat analysis of overall GLS, GWI, GCW, GWW, and GWE, ICC was 0.97 (95% CI, 0.947–0.990), 0.892 (95% CI, 0.753–0.953), 0.927 (95% CI, 0.830–0.968), 0.834 (95% CI, 0.618–0.928), and 0.877 (95% CI, 0.708–0.947), respectively (all *P* = 0.000).

## Discussion

To our knowledge, this is the largest prospective study to evaluate the reliability of the index beat method for assessing LV MW in AF patients across the full range of LVEF. We found that LV GWI, GCW, GWW, and GWE derived from the index beat closely matched the corresponding 10-beat average values and showed excellent reproducibility.

AF is characterized by irregular ventricular rhythm and beat-to-beat variation in ventricular contractility (4). Our study is unique in that it overcomes these limitations and extends the use of overall GLS and LV MW evaluation to the AF population, while also demonstrating a less time-consuming approach. Overall GLS derived from 17 LV segments has been validated as a robust measure of LV global systolic function, offering greater sensitivity than either LVEF or apical four-chamber GLS alone (6, 21, 22). However, in AF patients, data on 17-segment GLS have been scarce. Lee et al. (15) assessed LV GLS in AF patients but only analyzed the apical four-chamber view, covering six LV segments. Bunting et al. (23) measured overall GLS in AF using conventional 2DE, acquiring apical two-, three-, and four-chamber views separately, and then averaging of the sectional GLS values. Given the beat-to-beat variability in AF, such summation and averaging may fail to represent the true contractile status of any single beat (24). Our study addresses this challenge by using triplane echocardiography, which enables simultaneous acquisition of apical two-, three-, and four-chamber views with a single probe, allowing precise overall GLS measurement for each heartbeat. As MW is derived from overall GLS, this approach is feasible for extended use in AF. The present study provides robust supporting evidence.

Non-invasive MW indices are novel LV systolic function parameters incorporating LV afterload into GLS analysis (20, 25), with incremental diagnostic and prognostic value in multiple clinical scenarios (9, 25). However, in AF patients confronted with the same irregular ventricular rhythm problem, few studies have reported its use. Liu et al. (11) quantified MW in 51 patients with persistent AF; however, in their study, the apical views were acquired separately. Thus, the accuracy of the global value for each beat remains uncertain. To our knowledge, the present study is the first to assess LV MW with precise heartbeats in AF.

It is generally accepted that, in patients with AF, multiple measurements with subsequent averaging represent the standard approach for quantifying LV systolic function parameters (13). Nonetheless, evidence has shown that, to achieve an estimation of cardiac output with a variability of <2% compared with the mean of four beats in sinus rhythm, the number of beats required in AF is approximately three times greater (26). This is undoubtedly time-consuming for daily practice. Previous studies have validated the reliability and reproducibility of the index beat method for evaluating LV volume change rate (EF), intracardiac pressure change, aortic flow velocity and stroke distance, as well as GLS of the apical four-chamber view (14–17, 19, 27–29). Our study confirmed the reliability and reproducibility of the index beat method compared to the 10-beat average for quantifying overall GLS and global MW in AF. The present study showed higher GWI and GWE, and lower GWW using the index beat approach. These inter-method differences, although statistically significant, were small. Strong consistency was also supported by high ICC values. Further research including larger population remains warranted.

A previous study evaluating the index beat method defined the index beat as the beat with R-R intervals >500 ms (15). In contrast, our results show that measurements derived from the index beat with R-R intervals <500 ms can also be representative. In our cohort, seven patients had index beats with R-R intervals in the range of 384.6–491.8 ms, which produced representative results. This finding is clinically significant, as rapid heart rates and short R-R intervals are common in AF patients.

## Limitations

First, nearly one-third of patients did not demonstrate an eligible beat during the initial evaluation. Although re-analysis of additional triplane datasets improved the detection rate, this process was more time-consuming. With future advancements in machine learning and algorithms, real-time calculation of cardiac cycle length and automatic identification of the index beat may become feasible. Second, at present, only GE echocardiographic systems provide software capable of performing 2D STE in triplane datasets or MW analysis (20), which may limit generalizability across different commercial platforms. Nonetheless, as software capabilities evolve, more commercially available analysis tools can be anticipated. Third, this is a single-center study with a relatively small sample size; however, the number of patients was sufficient to achieve 90% power and a 99% concordance correlation coefficient. Besides, our findings in a heterogeneous AF population provide supportive evidence that may enhance the real-world applicability of the index beat method.

## Conclusion

In patients with AF undergoing echocardiography, global MW indices derived from the index beat were comparable with those calculated from 10 consecutive beats, demonstrating good reproducibility. Therefore, this approach offers a reliable method for advanced quantification of LV global systolic function in AF. Future multicenter studies with larger populations, broader workstation compatibility, and more advanced software are warranted to further validate and expand its clinical utility.

## Data Availability

The raw data supporting the conclusions of this article will be made available by the authors, without undue reservation.
